# Keeping the shape of plant tissue for visualizing metabolite features in segmentation and correlation analysis of imaging mass spectrometry in *Asparagus officinalis*

**DOI:** 10.1007/s11306-019-1486-5

**Published:** 2019-02-14

**Authors:** Ryo Nakabayashi, Kei Hashimoto, Kiminori Toyooka, Kazuki Saito

**Affiliations:** 1 RIKEN Center for Sustainable Resource Science, 1-7-22 Suehiro-cho, Tsurumi-ku, Yokohama, 230-0045 Japan; 2 Graduate School of Pharmaceutical Sciences, Chiba University, 1-8-1 Inohana, Chuo-ku, Chiba, 260-8675 Japan

**Keywords:** Metabolomics, Imaging mass spectrometry, Liquid chromatography−tandem mass spectrometry, Specialized metabolite, *Asparagus officinalis*

## Abstract

**Introduction:**

Matrix-assisted laser desorption/ionization−imaging mass spectrometry (MALDI−IMS) is a powerful approach for visualizing the localization of metabolites.

**Objectives:**

A method to keep the shape of plant tissue needs to be developed for MALDI−IMS.

**Methods:**

The method was developed using transfer tape and double-sided conductive tape.

**Results:**

MALDI−IMS analysis using the developed method enabled to perform segmentation and correlation analysis of mass features.

**Conclusion:**

This proof-of-concept study showed that rutin localizes in the epidermis, developing tissue, and protoxylem in *Asparagus officinalis*.

**Electronic supplementary material:**

The online version of this article (10.1007/s11306-019-1486-5) contains supplementary material, which is available to authorized users.

## Introduction

Specialized metabolites (previously called secondary metabolites) are significant natural products that are associated with certain species and accumulate in specific tissues and organs of plants. Previously, these metabolites were recognized as the byproducts of primary metabolites and were considered irrelevant (Hartmann 2007). However, recent phytochemical genomics studies have shown that they have important biological functions (Field et al. 2006; Massalha et al. 2017; Tohge et al. 2018). Comparative analysis of transcriptomics and metabolomics in transformants/mutants, which over-accumulate or lack certain metabolites by editing biosynthetic genes, can be used for identifying the functions of metabolites in plants (Nakabayashi et al. 2014). To identify biosynthetic genes responsible for specialized metabolites, understanding the association of metabolite accumulation with gene expression at certain parts is important (Saito 2013). However, this step for identifying the localization of the metabolites is time consuming. Therefore, new approaches need to be developed to reduce the time required.

Matrix-assisted laser desorption/ionization−imaging mass spectrometry (MALDI−IMS) is a powerful approach for visualizing the localization of metabolites and is used in sections of organisms (Dong et al. 2016; Fujimura and Miura 2014; Lee et al. 2012; Sarabia et al. 2018; Sturtevant et al. 2016). Recently, this approach has been applied to identify the localization of specialized metabolites in plants (Enomoto et al. 2018; Jarvis et al. 2017; Li et al. 2014; Shiono et al. 2017). One of the major challenges of MALDI−IMS is to keep plant tissue shapes during the preparation of sections. Plant tissues contain large amounts of water. A freeze-dried process is necessary to remove the water content from sections placed on glass slides. This process results in peeling the section from the slide using typical methods. Overcoming this problem may improve regional analysis via MALDI−IMS.

In the present study, a method named NakaMi was developed for the preparation of plant sections. As a proof of concept, this method was then applied to MALDI−IMS analysis on cross section in *Asparagus officinalis* (green asparagus), which is one of the staple crops in the world. A segmentation analysis showed that rutin (quercetin 3-*O*-rutinoside) localizes in the epidermis and developing stem tissue. Liquid chromatography−tandem mass spectrometry (LC−MS/MS) using the authentic standard compound supported the MALDI−IMS analysis result. The IMS analysis suggested that rutin accumulates in the protoxylem of *Asparagus*. A correlation analysis showed that the ion derived from rutin was correlated with the mass features of other metabolites.

## Materials and methods

### Chemicals

The chemicals used in this study are listed in Supplementary Table 1.

### Plant materials

Spears of *Asparagus officinalis* (green asparagus) were harvested at the end of April, 2018 from the Medicinal Plant Garden of Hoshi University, Japan.

### Preparing section

To prepare sections for MALDI−IMS analysis, a fresh *Asparagus* spear was transversely cut (for an approximate thickness of 10 mm) with a razor, then embedded with a reagent (Surgipath FSC22: Leica Microsystems, Germany), and frozen in a − 75 °C acetone bath (Histo-Tek Pino: Sakura Finetek Japan Co., Ltd., Tokyo, Japan). The frozen sample block was placed on a cryostat specimen disk and was cut with the knife blade until the desired tissue surface appeared. Transfer tape (Adhesive Tape Windows, Leica Microsystems, Germany) was placed on the face of the block to obtain sections (each with a thickness of 20 µm) in the CM3050S cryostat (Leica Microsystems, Germany). These sections were transferred to conductive Cu tape (double-sided, No. 796) (TERAOKA SEISAKUSHO, Co. Ltd.) on a glass slide (ITO coating, Bruker Daltonik GmbH). The section on the glass slide was freeze-dried overnight at − 30 °C in the cryostat. For light microscopy, the frozen sections were stained with 0.05% toluidine-blue O solution for 1 min and washed with distilled water. The micrographs were acquired with a BX51 microscope equipped with a digital camera (Olympus cellSens and DP26).

### MALDI−IMS analysis

A 2,5-dihydroxybenzoic acid (DHB) matrix solution (Supplementary Table 1) was sprayed on the prepared section that was on the glass slide using ImagePrep (Bruker Daltonik GmbH) running at the default parameters. The freeze-dried section with the matrix was analyzed in SolariX 7.0 T instrument (Bruker Daltonik GmbH) for Fourier transform ion cyclotron resonance−mass spectrometry. The MALDI parameters are as follows: geometry, MTP 384 ground steel; plate offset, 100.0 V; deflector plate, 200.0 V; laser power, 50.0%; laser shots, 200; frequency, 2000 Hz; laser focus, small; raster width, 60 µm. The analytical conditions for the IMS analysis were identical to those for MALDI−MS analysis (Supplementary Methods).

The segmentation analysis was performed with the SCiLS Lab software (version 2019a). The mass features in the MALDI−IMS data (15,732 data points) were divided into seven groups by the bisecting *k*-means clustering (parameters: metric, Manhattan; minimal interval width, ± 1.8111 mDa). The three groups of ions (purple for the ground tissue, yellow for the vascular bundles, and green for the epidermis and developing tissue) are listed in Supplementary Data 1.

Pearson’s correlation analysis was performed using the SCiLS Lab. The ion at *m*/*z* 633.1425 observed as [M + Na]^+^ from rutin was calculated with 3,243 mass features in half of the cross section (Supplementary Figure 1). A minimal interval width was set at ± 1.8111 mDa. Ions with correlation coefficient greater than 0.4 are listed in Supplementary Data 2.

## Results and discussion

To determine which matrix reagent is appropriate for detecting wide range of metabolites, we first performed the screening of matrix reagents in 36 metabolites that belongs to 12 metabolite types (alkaloid, anthocyanidin, anthocyanin, flavonoid aglycone, flavonoid glycoside, glucosinolate, lignan, phenolamide/hydroxycinnamic acid amide, phenylpropanoid/coumarin, saponin/glycoalkaloid aglycone, saponin/glycoalkaloid, and sulfur-containing metabolite) using four matrix reagents, including α-cyano-4-hydroxycinnamic acid (CHCA), 1,5-diaminonaphthalene, DHB, and 3-hydroxypicolinic acid, via MALDI−MS analysis (Supplementary Methods and Supplementary Table 2). Three compounds per metabolite type were selected, resulting in a total of 36 authentic standard compounds. The results suggest that CHCA and DHB are appropriate for detecting metabolites at a high sensitivity in positive ion mode of MALDI−IMS analysis. Notably, the signal intensities for DHB tended to be higher than those observed for CHCA. Negative ion mode that requires different parameters and reagents was out of scope in this study.

**Fig. 1:**
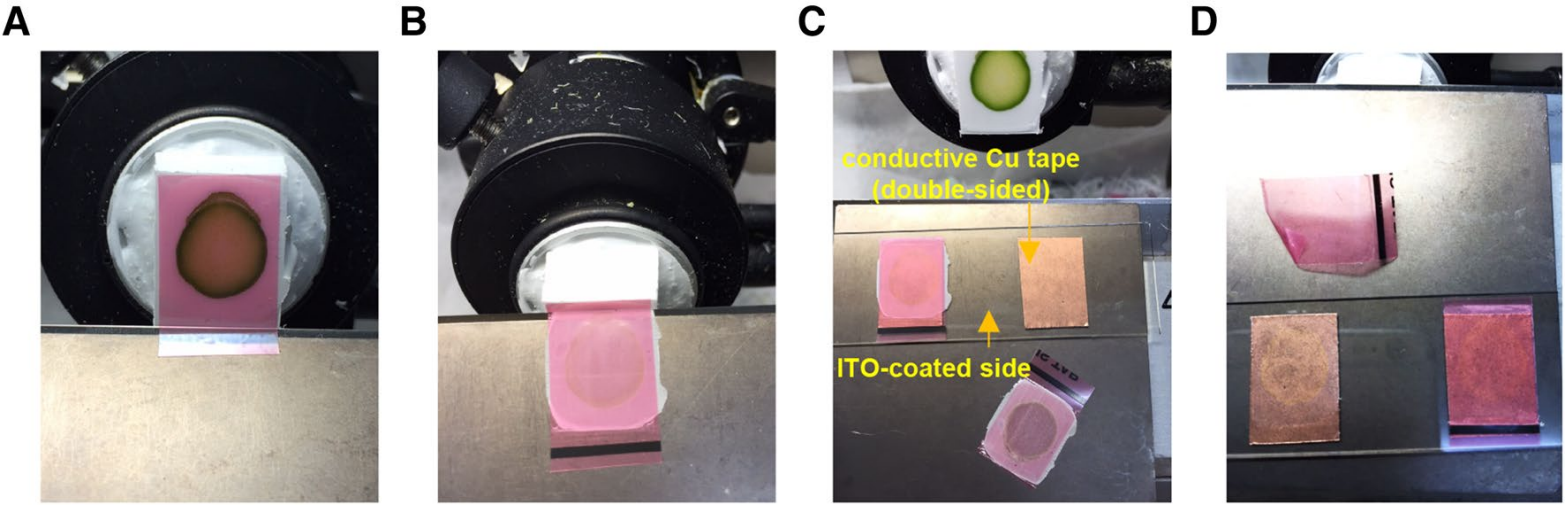
Procedure of preparing plant sections for MALDI−IMS in this study. **A** The transfer tape (pink) was put on top of the block of the embedding reagent with the plant sample in the chilled cryostat. **B** Sections were cut with the transfer tape. **C** The transfer tape was put on the conductive tape and dapped by finger. **D** The transfer tape was gently removed by hand

A method using transfer and conductive tape (termed the NakaMi method) was developed to keep the shape of the plant tissue on glass slides for MALDI−IMS analysis. All processes were performed in a chilled cryostat. The section was placed on the conductive tape (marketed product, not for MALDI−IMS). Subsequently, the transfer tape was gently removed by hand (Fig. 1). The plant tissues were not removed from the conductive tape after spraying on the matrix reagent and MALDI−IMS analysis.

**Fig. 2:**
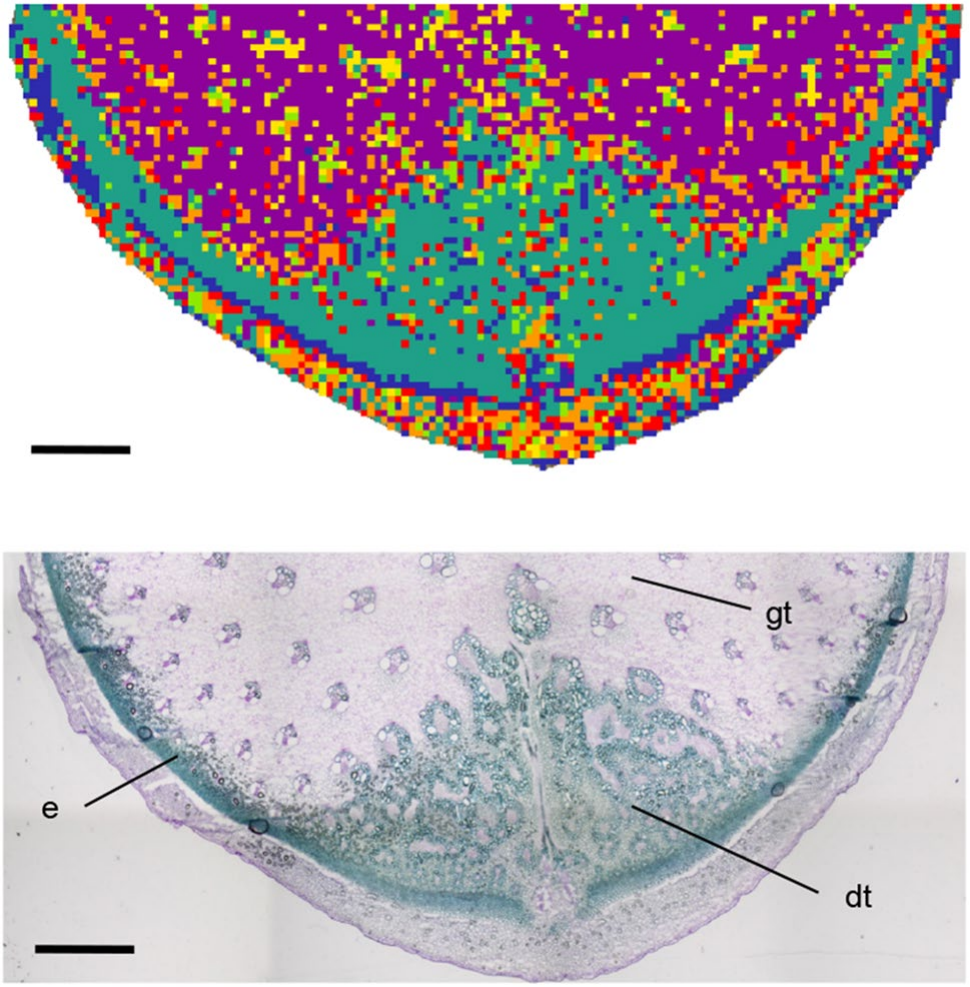
Segmentation of detected mass features. Upper. The map highlighting the result in the segmentation analysis. All mass features were divided into seven colored groups (purple, red, orange, yellow, light green, blue, and green) in this case. Lower. Light microscopy in the sections stained using toluidine-blue O. *dt* developing tissue, *e* epidermis, *gt* ground tissue. Bar indicates 1 mm

For the IMS analysis, DHB was selected to detect different types of metabolites in *Asparagus officinalis* (green asparagus). To understand the pattern of mass features of detected metabolites, the segmentation analysis was performed using data from the cross section. The whole data comprised 15,732 data points; however, for data analysis, only half (7916 data points) were used for reducing the number of data points (Fig. 2). The colored map clearly showed that tissue-redundant metabolites existed in the section. The 11,365 mass features in three of the seven groups (green, yellow, and purple) subjected to segmentation analysis are provided in Supplementary Data 1.

**Fig. 3:**
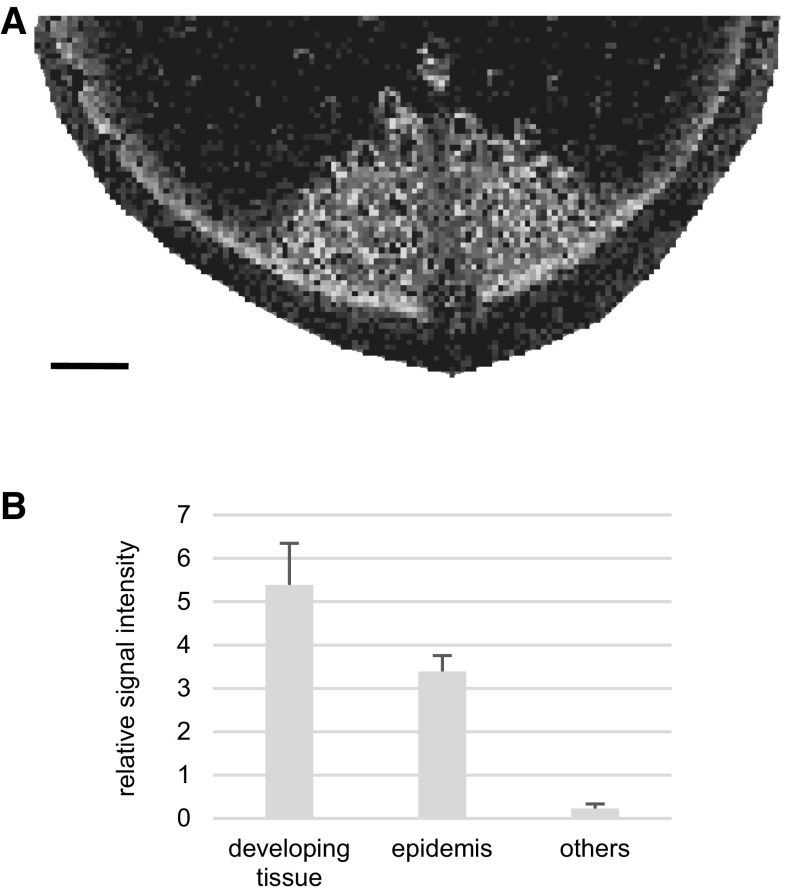
Identifying the localization of rutin. **A** The localization of rutin was visualized with *m*/*z* 633.1425 ± 1.8111 as [M + Na]^+^ form on the cross section. Bar indicates 1 mm. **B** Comparing the relative signal intensity of rutin in liquid chromatography–tandem mass spectrometry. The signal intensity of rutin was divided with that of the internal standard lidocaine in each sample. Others include the vascular and ground tissues. Bar indicates standard deviation (n = 3)

**Fig. 4:**
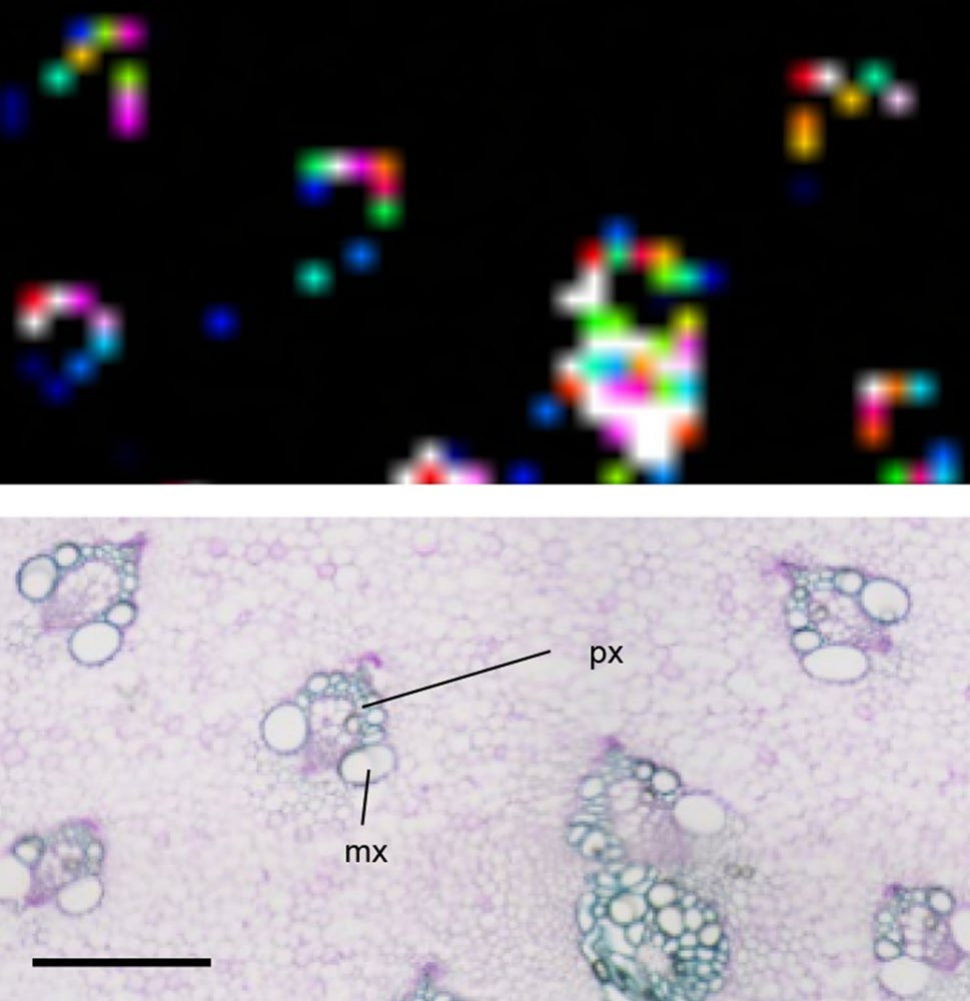
The localization of rutin in the protoxylem characterized by the IMS analysis. Upper. The localization of rutin with the ion *m*/*z* 633.1425 ± 1.8111 as [M + Na]^+^. Lower. Light microscopy in the sections stained using toluidine-blue O. *mx* metaxylem, *px* protoxylem. Bar indicates 500 µm

Rutin is a health-promoting specialized metabolite with antioxidant activity (Butelli et al. 2008; Tohge et al. 2015). This metabolite accumulates in the epidermis of plants, e.g., in the leaves of *Zea mays* (Korte et al. 2015). In *Asparagus*, the localization of rutin has not been determined yet using MALDI−IMS. The IMS analysis showed that the ion at *m*/*z* 633.1425 was determined to be [M + Na]^+^ from rutin, which was distributed in the green group of the segmentation map. Light microscopy showed that the green represents the epidermis and developing stem tissue (Figs. 2 and 3a). To identify rutin, LC−MS/MS analysis was performed using the authentic standard compound (Supplementary Figure 2 and Supplementary Methods), and it showed that the amount of rutin in the developing tissue and epidermis was more than that in the others’ part including the ground tissue and vascular bundles (Fig. 3b). In addition, the IMS analysis showed that rutin accumulates in the protoxylem (Fig. 4). Previous research identified the localization of flavonol monoglycosides, including quercetin-3-*O*-β-glucoside, in xylem parenchyma cells (Kasuga et al. 2008).

The localization of rutin in the epidermis in this study is a rational finding. Surprisingly, rutin accumulated in the developing tissue that was not exposed to sunlight yet. The following hypotheses for the function of rutin in developing tissue are considered: (1) rutin may provide the young cells of the stem an instantaneous mitigation of the production of reactive oxygen species (ROS) against its unavoidable future exposure to sunlight, due to its further outgrowth, (2) rutin may mitigate the production of ROS in the developing tissue (Manzano et al. 2014), or (3) xylem parenchyma cells accumulating rutin are differentiated in the developing tissue. To confirm these hypotheses, further studies need to be performed. For instance, correlation analysis is a powerful tool to understand co-localized metabolites in the protoxylem. Data from the region of the ground tissue and vascular bundles were used for the analysis (Supplementary Figure 1). This showed that the observed ion at *m*/*z* 633.1425 was correlated with 177 mass features (Pearson correlation coefficient PCC, ≥ 0.4) (Supplementary Data 2). The correlation coefficient of monoisotopic ion (*m*/*z* 633.1425) to its isotopic ion including ^13^C (*m*/*z* 634.1455) was 0.41. The IMS data included data points in which both the monoisotopic and isotopic ion were detected in higher signal intensity, and only monoisotopic ion was detected in lower signal intensity. The bias to detecting only the monoisotopic ion resulted in the lower value. Based on this result, the highly correlated ions with the ion at *m*/*z* 633.1425 can be candidates for chemical assignment in the future in another study.

Identification of the metabolites may help to understand the function of rutin in the protoxylem. Integrated metabolomics with transcriptomics in the protoxylem can provide the associations of genes with rutin. The function of rutin may be predicted using annotations of genes associated with the metabolites. It is known that *Asparagus* is a transformable plant (Bytebier et al. 1987). Comparative analysis of the wild type gene and a transformant that lacks rutin can help directly understand its function.

The chemical diversity of the metabolome in the plant kingdom is vast. To probe the metabolome, polyhedral approaches are required. For example, some metabolites have been specifically detected via either MALDI−MS or electrospray ionization−MS (Nakabayashi et al. 2017). Additionally, analysis of these types of metabolites via IMS is becoming more common in the plant science field (Boughton et al. 2016; Etalo et al. 2015). The combination of both approaches is necessary for the comprehensive analysis of the localization of such metabolites.

## Electronic supplementary material

Below is the link to the electronic supplementary material.

Supplementary material 1 (DOCX 17 KB)

Supplementary material 2 (XLSX 544 KB)

Supplementary material 3 (XLSX 14 KB)

Supplementary material 4 (PPTX 2410 KB)

Supplementary material 5 (XLSX 13 KB)

Supplementary material 6 (XLSX 16 KB)
