# Supplementary material for: Keeping the shape of plant tissue for visualizing metabolite features in segmentation and correlation analysis of imaging mass spectrometry in Asparagus officinalis
Source: Metabolomics. 2019 Feb 14;15(2):24. doi: 10.1007/s11306-019-1486-5 (PMC6394462; doi:10.1007/s11306-019-1486-5)
Supplement: Supplementary file 1 — Supplementary material 1 (DOCX 17 KB) [file 11306_2019_1486_MOESM1_ESM.docx]

**Keeping the shape of plant tissue for visualizing metabolite features in segmentation and correlation analysis of imaging mass spectrometry in *Asparagus officinalis***

Ryo Nakabayashi^1,*^, Kei Hashimoto^1^, Kiminori Toyooka^1^, and Kazuki Saito^1,2^

1 RIKEN Center for Sustainable Resource Science, 1-7-22 Suehiro-cho, Tsurumi-ku, Yokohama 230-0045, Japan

2 Graduate School of Pharmaceutical Sciences, Chiba University, 1-8-1 Inohana, Chuo-ku, Chiba 260-8675, Japan

ORCID: Ryo Nakabayashi (0000-0002-8674-0928)

* Corresponding author: Ryo Nakabayashi ([ryo.nakabayashi@riken.jp](mailto:ryo.nakabayashi@riken.jp))

**Supplementary Methods.**

**MALDI**−**MS analysis**

Solutions of the authentic standard compounds (100 mM, 0.2 μL) were dispensed into 384-well plates and mixed with a 2,5-dihydroxybenzoic acid (DHB) matrix reagent solution [30 mg/mL 50% MeOH including 0.2% trifluoroacetic acid (TFA), 0.2 μL]. The crystals obtained on the plate were analyzed by the FTICR−MS solariX 7.0 T (Bruker Daltonics) operated with the MALDI source. The Na(formate) solution (the mixture of 10% formate and 0.1N NaOH, 1:1) was analyzed for lockmass with the ESI source during the analysis of MALDI−MS or MALDI−IMS. The analytical conditions were as follows: mass range, *m/z* 200.64−1500.00; average scan, 1; accumulation, 0.650 s; polarity, positive; source quench, on; API high voltage, on; resolving power, 66,000 at 400 *m/z*; transient length, 0.4893 s; Mode (data storage: save reduced profile spectrum, on; reduced profile spectrum peak list, on; data reduction, 95%; auto calibration: online calibration, on; mode, single; reference mass, *m/z* 770.85088); API Source (API source: source, ESI; capillary, 4500 V, end plate offset, −500; source gas tune: nebulizer, 1.0 bar; dry gas, 2.0 l/min; dry temperature, 100 °C); Ion Transfer (source optics: capillary exit, 220 V; detector plate, 200 V; funnel 1, 150 V; skimmer 1, 20 V; funnel RF amplitude, 200 Vpp; octopole: frequency, 2 MHz; RF amplitude, 350 Vpp; quadrupole: Q1 mass, 200 *m/z*; collision cell: collision voltage, −1.0 V; DC extract bias, 0.9 V; RF frequency, 2 MHz; collision RF amplitude, 1500.0 Vpp; transfer optics: time of flight, 0.750 ms; frequency, 2 MHz; RF amplitude, 350.0 Vpp); Analyzer (infinity cell: transfer exit lens, −20.0 V; analyzer entrance, −10.0 V; side kick, 5.0 V; side kick offset, 0.0 V; front trap plate, 0.870 V; back trap plate, 0.915 V; sweep excitation power, 12.0%; multiple cell accumulations: ICR cell fills, 1).

**LC−MS/MS analysis**

The asparagus spears were manually separated to three parts, developing tissue, epidermis, and other parts. The parts were immediately lyophilized at −55 °C. Then, the freeze-dried samples were completely powdered. The powdered samples (three mg dried weight) were extracted with 500 µl of 80% MeOH containing 2.5 µM lidocaine per mg dry weight using a mixer mill with zirconia beads for 7 min at 18 Hz and 4 °C (MM300; Retsch). After centrifugation at 16,000 × *g* for 10 min, the supernatant was filtered using an HLB µElution plate (Waters). The 100 µL extract was transferred to a glass amber vial with a micro-insert (Agilent Technologies) for LC−MS/MS analysis. The extracts (1 µl) were analyzed using LC−QTOF−MS instrument (LC, Waters Acquity UPLC system; MS, Waters Xevo G2 Q-Tof). Analytical conditions were as follows LC: column, Acquity bridged ethyl hybrid (BEH) C18 (1.7 µm, 2.1 mm × 100 mm, Waters); solvent system, solvent A (water including 0.1% formic acid) and solvent B (acetonitrile including 0.1% formic acid); gradient program, 99.5%A/0.5%B at 0 min, 99.5%A/0.5%B at 0.1 min, 20%A/80%B at 10 min, 0.5%A/99.5%B at 10.1 min, 0.5%A/99.5%B at 12.0 min, 99.5%A/0.5%B at 12.1 min and 99.5%A/0.5%B at 15.0 min; flow rate, 0.3 ml/min at 0 min, 0.3 ml/min at 10 min, 0.4 ml/min at 10.1 min, 0.4 ml/min at 14.4 min and 0.3 ml/min at 14.5 min; column temperature, 40 °C; MS detection: polarity, positive; capillary voltage, +3.00 kV (positive); cone voltage, 25.0 V; source temperature, 120 °C; desolvation temperature, 450 °C; cone gas flow, 50 l/h; desolvation gas flow, 800 l/h; collision energy, 6 V; mass range, *m/z* 50–1500; scan duration, 0.1 sec; inter-scan delay, 0.014 sec; data acquisition, centroid mode; lockspray, leucine enkephalin; scan duration, 1.0 sec; inter-scan delay, 0.1 sec. MS/MS data was acquired as the following analytical conditions: (1) MS: polarity, positive; mass range, *m/z* 50–1500; scan duration, 0.1 sec; inter-scan delay, 0.014 sec; data acquisition, centroid mode and (2) MS/MS: polarity, positive/negative; mass range, *m/z* 50–1500; scan duration, 0.02 sec; inter-scan delay, 0.014 sec; data acquisition, centroid mode. In this mode, MS/MS spectra of the top 10 ions (> 1000 counts) in an MS scan were automatically obtained. If the ion intensity was less than 1000, MS/MS data acquisition was not performed and moved to of next top 10 ions. The LC−MS/MS analysis was performed in three biological replicates.
