# Supplementary material for: Keeping the shape of plant tissue for visualizing metabolite features in segmentation and correlation analysis of imaging mass spectrometry in Asparagus officinalis
Source: Metabolomics. 2019 Feb 14;15(2):24. doi: 10.1007/s11306-019-1486-5 (PMC6394462; doi:10.1007/s11306-019-1486-5)
Supplement: Supplementary file 4 — Supplementary material 4 (PPTX 2410 KB) [file 11306_2019_1486_MOESM4_ESM.pptx]

## Slide 1
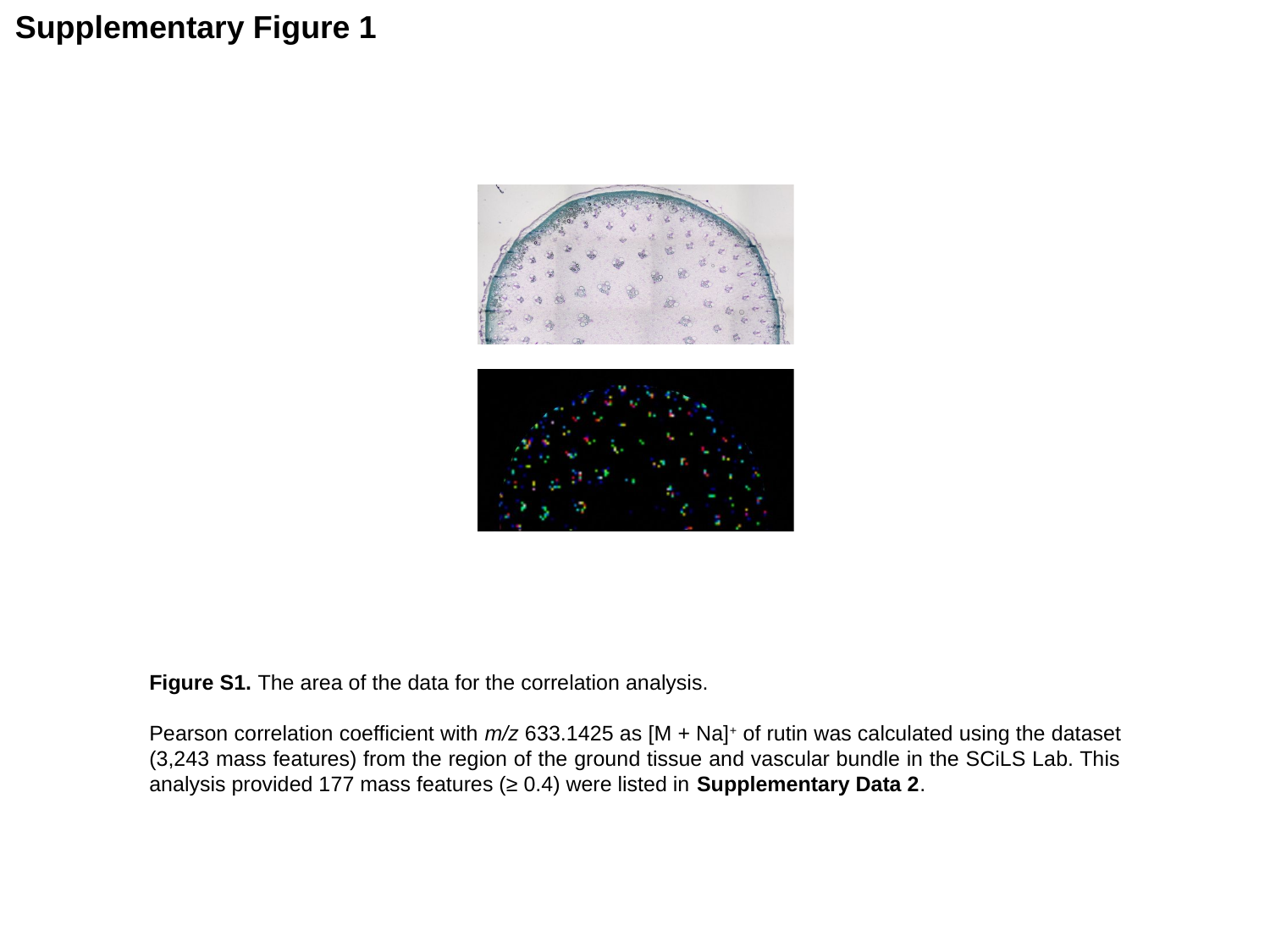

Supplementary Figure 1
Figure S1. The area of the data for the correlation analysis.
Pearson correlation coefficient with m/z 633.1425 as [M + Na]+ of rutin was calculated using the dataset (3,243 mass features) from the region of the ground tissue and vascular bundle in the SCiLS Lab. This analysis provided 177 mass features (≥ 0.4) were listed in Supplementary Data 2.

## Slide 2
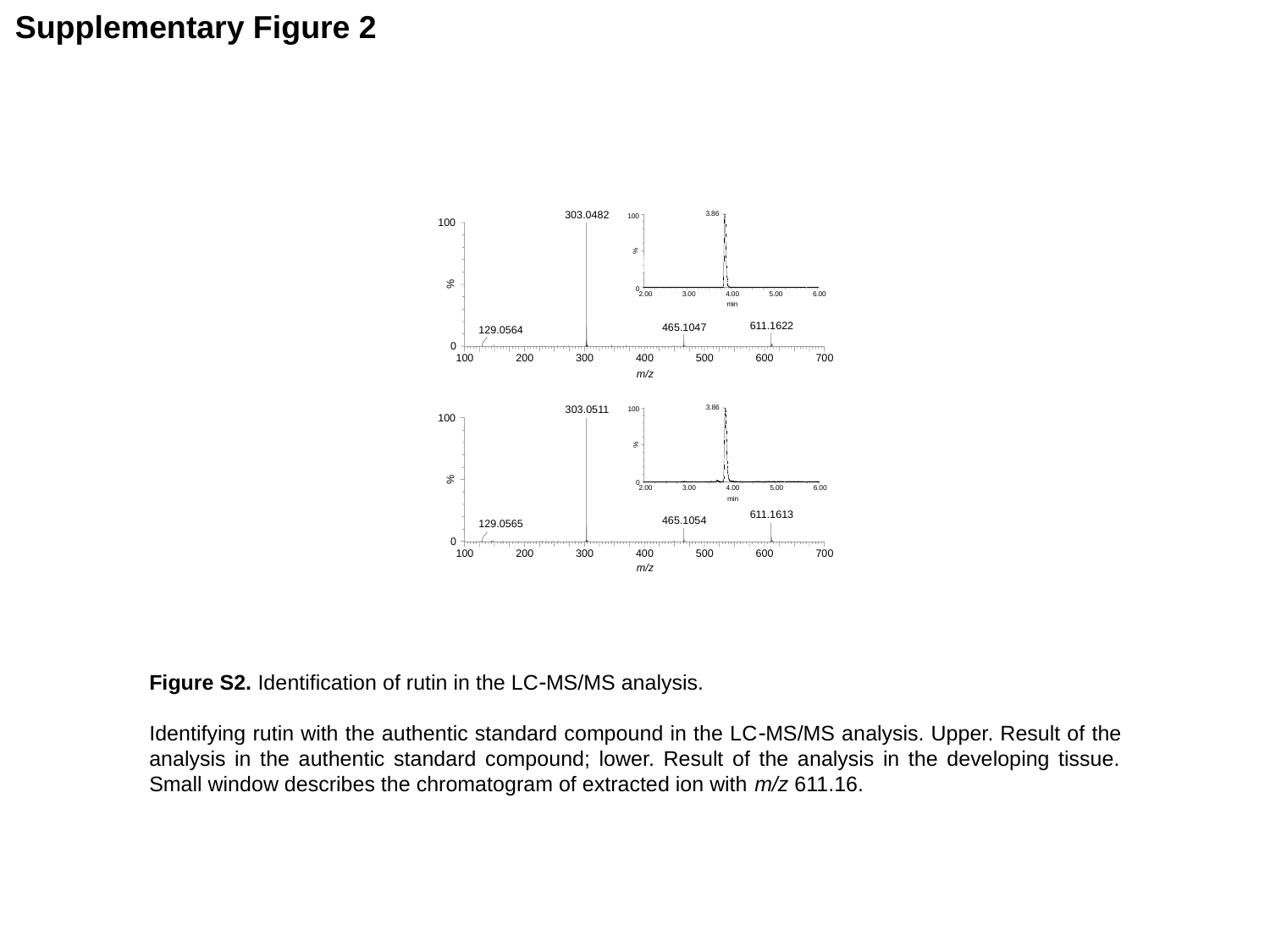

Supplementary Figure 2
100
%
0
100
200
300
400
500
600
700
m/z
303.0482
611.1622
465.1047
129.0564
3.86
100
%
0
2.00
3.00
4.00
5.00
6.00
min
303.0511
100
%
0
100
200
300
400
500
600
700
m/z
611.1613
465.1054
129.0565
3.86
100
%
0
2.00
3.00
4.00
5.00
6.00
min
Figure S2. Identification of rutin in the LC−MS/MS analysis.
Identifying rutin with the authentic standard compound in the LC−MS/MS analysis. Upper. Result of the analysis in the authentic standard compound; lower. Result of the analysis in the developing tissue. Small window describes the chromatogram of extracted ion with m/z 611.16.
